# The prevalence of EBV and CMV DNA in epithelial ovarian cancer

**DOI:** 10.1186/s13027-019-0223-z

**Published:** 2019-02-26

**Authors:** Kasper Ingerslev, Estrid Høgdall, Wojciech Skovrider-Ruminski, Tine Henrichsen Schnack, Marianne Lidang, Claus Høgdall, Jan Blaakaer

**Affiliations:** 1 Department of Gynecology, Odense University Hospital, Sdr. Boulevard 29, 5000 Odense C, Denmark; 2 Department of Pathology, Herlev Hospital, Herlev Ringvej 75, 2730 Herlev, Denmark; 3 Department of Gynecology, Rigshospitalet, Copenhagen University Hospital, Blegdamsvej 9, 2100 Copenhagen Ø, Denmark

**Keywords:** Epithelial ovarian cancer, Viral carcinogenesis, Cytomegalovirus, Epstein-Barr virus

## Abstract

**Background:**

The underlying cause of epithelial ovarian cancer (EOC) is unknown. It has been theorized that infectious agents could contribute to ovarian tumorigenesis.

**Objective:**

To investigate the potential role of oncogenic viral infection in EOC, we examined the prevalence of Epstein-Barr Virus (EBV) DNA and cytomegalovirus (CMV) DNA in EOC tissue samples.

**Methods:**

Formalin-fixed, paraffin-imbedded (FFPE) tumor tissue samples from 198 patients included in the Danish Pelvic Mass Study were studied: 163 with serous adenocarcinomas, 15 with endometrioid adenocarcinomas, 11 with mucinous adenocarcinomas, and nine with clear-cell carcinomas. For controls in the EBV analysis, we used 176 tissue samples from patients diagnosed with benign mucinous cystadenomas. EBV and CMV genotyping was performed by real-time polymerase chain reaction with CMV and EBV CE-IVD approved kits. In-situ hybridization (ISH) was performed on the EBV positive samples.

**Results:**

Sufficient DNA material was obtained in 191 and 174 tissue samples from cases and controls, respectively. Ten of 191 case samples (5.2%) and one of 174 control samples (0.5%) were positive for EBV DNA (*P* value = 0.011). CMV DNA was detected in only one case sample (0.5%). ISH confirmed that three of the samples were of stromal origin, while the remaining seven tested negative for EBV.

**Conclusions:**

This study is the first to demonstrate a higher prevalence of EBV DNA in tissue samples from patients with EOC than in a benign control group. However, the cellular origin of seven of the samples could not be determined by ISH analysis. Our study did not support an association between CMV and EOC.

**Electronic supplementary material:**

The online version of this article (10.1186/s13027-019-0223-z) contains supplementary material, which is available to authorized users.

## Introduction

Epithelial ovarian cancer (EOC) has a poor prognosis, with a global 5-year age-standardized survival rate of 30–40% [1]. Patients often present with advanced-stage disease due to the absence of effective screening methods and the scarceness of symptoms [2]. EOC is subdivided into serous (high-grade and low-grade adenocarcinomas), mucinous, clear-cell, and endometrioid carcinomas, the serous type being by far the most frequent [3]. Data suggest that EOC subtypes are separate entities, characterized by distinct histopathologic, prognostic, and molecular profiles [4–6]. Some of the subtypes may have an extra-ovarian origin, and recently, serous tubal intraepithelial carcinomas (STICS) have been shown to be present in the tubal fimbria and could represent precursor lesions of serous EOC [7, 8].

Germline mutations, most notably BRCA1/2, can account for up to 20% of EOC cases [9]. Other predisposing factors are nulliparity, infertility, and endometriosis, whereas parity, contraceptive pills, hysterectomy, and tubal ligation are known to reduce EOC risk [10, 11]. The predominant hypothesis on ovarian carcinogenesis suggests that incessant ovulation and the continuous cycle of ovarian rupture and inflammation causes the release of inflammatory mediators and reactive oxygen species that could cause mutagenic DNA damage [12]. However, our current knowledge of ovarian carcinogenesis is deficient, compromising advances in early detection measures and prevention.

The female anatomy allows access to the internal genitals through the genital tract. This is evident in pelvic inflammatory disease (PID), where an ascending infection can cause permanent damage to the fallopian tubes and compromise fertility [13]. Moreover, some studies have reported an increased risk of EOC in patients with prior PID [14, 15]. Thus, infectious agents could be involved in ovarian carcinogenesis.

Cytomegalovirus (CMV) and Epstein-Barr virus (EBV) seroprevalence rates are high in the global population [16, 17]. The viruses can establish life-long latency in human cells and thereby evade the host immune response [18, 19]. EBV is implicated in the development of nasopharyngeal and gastric carcinomas as well as in a range of lymphoproliferative disorders [20–22].

CMV contains several onco-proteins with the potential to cause immune evasion and affect human cell cycle progression and angiogenesis [23]. CMV is suspected to elicit an onco-modulatory effect by sustaining and promoting a tumor-friendly milieu and thus aiding cancer progression and metastasis [24]. The virus has been detected in gliomas, colorectal carcinomas, hepatocellular carcinomas, and breast cancers [25–28].

To elucidate the potential role of EBV and CMV in ovarian carcinogenesis, we investigated the prevalence of both viruses in epithelial EOC tissue samples and compared findings to those obtained in a benign control group.

## Material

The tissue samples studied originated from the Danish Pelvic Mass study, which is a continuing and national sampling of serum and tissue samples. The inclusion criterion was referral for surgery to a specialized center on suspicion of EOC. Exclusion criteria were relapse of prior malignant disease, previous neoadjuvant chemotherapy (NACT), or synchronous cancer other than EOC.

Approval by the Danish National Committee on Health Research Ethics was obtained (KF01–227/03 and KF01–143/04, H-3-2010-022), and all patients consented orally and in written statements prior to enrollment in the Pelvic Mass study. The rate of acceptance was 95% among eligible patients.

**Fig. 1:**
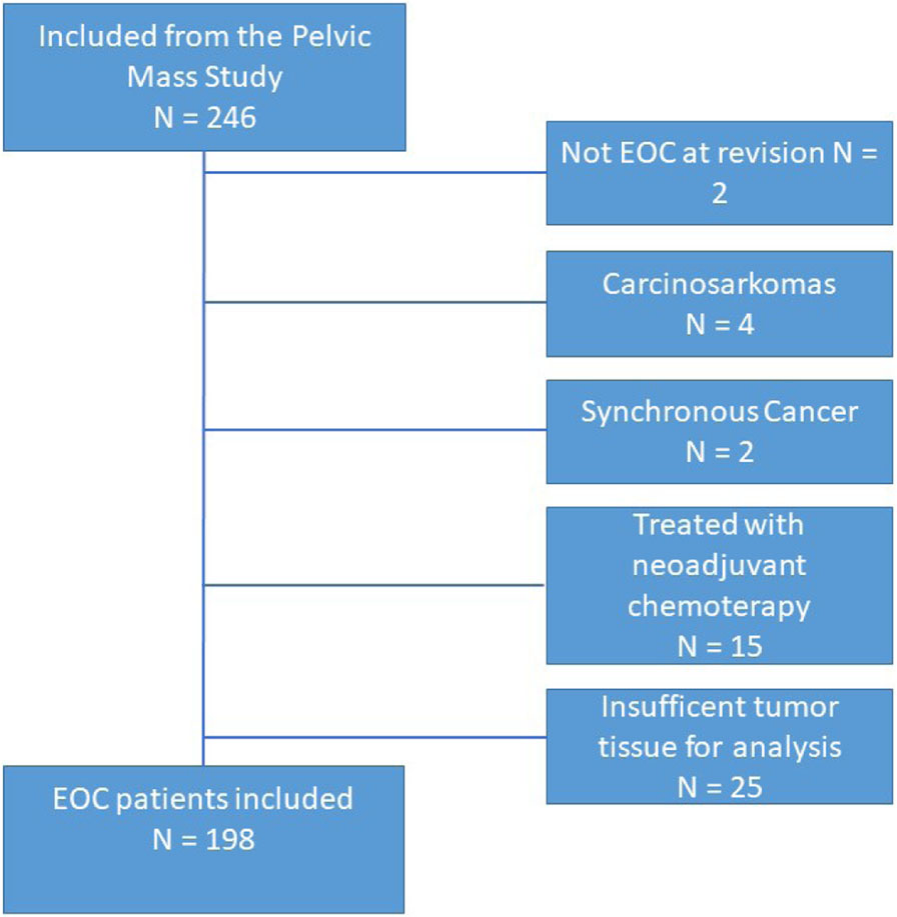
Inclusion of case patients. *EOC* Epithelial ovarian cancer

For the present study, formalin-fixed and paraffin-embedded (FFPE) EOC tumor samples were obtained from 246 patients with EOC consecutively included from Copenhagen University Hospital, Rigshospitalet, and Herlev Hospital in the time span 2004–2010. After revision of the samples, 48 patients had to be excluded for the reasons given in Fig. 1.

FFPE samples from 176 patients with benign ovarian tumors were included as a control group. These patients underwent surgery due to suspicion of EOC. However, the final pathology report revealed benign mucinous ovarian tumors in all controls. The control group was used exclusively for the EBV analysis.

The Danish Gynecologic Cancer Database was used to document and store patient data, while the tissue specimens were sampled perioperatively and preserved in the Danish Cancer Biobank [29].

## Methods

### DNA based analyses

The FFPE tissue specimens were assessed by a pathologist, and representative areas were selected and sampled with a 2-milimeter biopsy punch.

Qiagen® Qiacube equipment was used for DNA extraction, and the concentration of DNA was subsequently measured on Nanodrop® and diluted to 5 ng/μl.

The following DNA analyses were carried out in compliance with the manufacturer’s instructions [30, 31]. For the CMV analysis, genotyping was performed with the Altona Realstar® CMV, CE-IVD approved kit with sensitivity of 99.04% and specificity of 100%.

For the EBV analyses of the malignant samples and the benign controls, genotyping was done with the Altona Realstar® EBV, CE-IVD approved kit. For the detection of EBV-specific DNA, the analytical sensitivity is 1.1 copies/μl; 95% confidence interval (CI): [0.578–3.253 copies/μl.

The Roche®LC480 Lightcycler was used for real-time polymerase chain reaction (PCR) analysis.

The cycling parameters were 95 °C for 10 min continued by 45 cycles at 95 °C for 15 s, 58 °C for 1 min. During each PCR reaction, a 10-μl sample template or controls were used. Each operation included four positive controls and one negative control.

### ISH

From the tissue samples found positive by PCR, ISH was performed. Tissue slides of 2.5–3 /μm were used. Briefly, after deparaffinization, sections were digested with proteinase K at 37 °C for 30 min, dehydrated and dried. Hybridization with digoxigenin-labeled probes was then performed according to manufacturer’s instruction. BOND ISH probes (EBER probe ASR, ready-to-use Novocastra PB0589) were used.

An EBV-positive control, simultaneously stained by the same method, was included.

## Statistical analyses

STATA statistical software version 14.2 was used for the statistical analysis.

Logistic regression was used to estimate odds ratios with 95% CIs by comparing the case group to the control group. The *p* value was calculated using 2-sided Fisher’s exact test, and a *p* value below 0.05 was considered significant.

## Results

Table 1 lists the tumor histopathology, age, and International Federation of Gynecology and Obstetrics (FIGO) stage distribution of the 198 case patients. The median age of the EBV controls was 55 years (range 77) and 64 years (range 58) in the case patients. No other variables were available for the control group.

**Table 1:** Histopathology, stage, and age of included case patients

	EOC included *N* = 198
Age, median (range)	64 (58)
FIGO Tumor stage	
Stage I, n (%)	31 (15.7%)
Stage II, n (%)	21 (10.6%)
Stage III, n (%)	120 (60.1%)
Stage IV, n (%)	26 (13.1%)
Histopathology	
*Serous adenocarcinoma,* n *(%)*	163 (82.3%)
*Mucinous adenocarcinoma,* n *(%)*	11 (5.6%)
*Endometrioid adenocarcinoma,* n *(%)*	15 (7.6%)
*Clear Cell carcinoma,* n *(%)*	9 (4.5%)

*EOC* Epithelial ovarian cancer

*FIGO* International Federation of Gynecology and Obstetrics

For real-time PCR analysis, seven samples were excluded due to low amounts of DNA.

Ten of the remaining 191 EOC samples were positive for EBV DNA (5.2%) as was one (0.5%) of the controls. EBV DNA positivity in the control group was confirmed by in ISH and immunohistochemistry. The distribution of EBV-positive samples along with EOC histologic grade, subtype, and FIGO stage is given in Table 2.

**Table 2:** Characteristics of EBV-positive cases

No.	Age	FIGO stage	Histologic grade	EOC subtype
1	80	IIb	2	Serous
2	60	Ia	3	Serous
3	81	IV	3	Serous
4	52	IV	3	Serous
5	72	IIIc	3	Serous
6	75	IIIc	2	Serous
7	86	IIIc	3	Serous
8	81	IIIc	3	Serous
9	64	IIc	2	Serous
10	53	IV	3	Serous

*EOC* Epithelial ovarian cancer

*FIGO* International Federation of Gynecology and Obstetrics

The cycle threshold values ranged between 33 and 37, indicating weak infections with a low number of viral copies. To test the validity of the results, the analysis was repeated in four runs with identical, positive results.

The prevalence of EBV DNA was significantly higher in the EOC samples than in the benign ovarian tumors (Table 3). The EBV positive samples were subsequently tested by ISH to determine whether the signal originated from stromal cells or invading leukocytes. For three out of the 10 samples a positive signal originating from both stromal cells and leukocytes was confirmed. The remaining 7 tissue samples were negative for EBV using the ISH analysis.

**Table 3:** Results of EBV PCR analysis in case and control patients

	EBV positive no (%)	EBV negative no (%)	Total no (%)
Cases	10 (5.2)	181 (94.7)	191 (100)
Controls	1 (0.5)	173 (99.4)	174 (100)
total	11 (3.0)	354 (96.9)	365 (100)
	Odds ratio: 9.55, 95% Confidence interval: [1.32–417.06] 2-sided Fischer’s exact test *p* value = 0.011		

CMV DNA was detected in one (0.5%) case sample only, and due to the low prevalence, results were not compared to a control group.

## Discussion

This study is the first to report a significantly higher prevalence of EBV in EOC tissue samples than in samples from a benign control group of ovarian benign tumors.

The strengths of the present study were the meticulous retesting of results and the structured information on the patients in the case cohort. Another strength was the use of consecutive sampling of case and control patients, which reduces the risk of sample selection bias. The study also had some potential limitations. The DNA material contained in FFPE tissue samples is prone to degradation and fragmentation, which can potentially compromise results [32]. However, we have previously demonstrated HPV DNA in 90% of anal cancers using a comparable FFPE material and similar PCR-based techniques [33].

Under optimal conditions, more variables should have been available to allow for adjustment of potential confounding factors. Age distribution was available for both case and control groups, and we found that the controls were younger than the case patients (median 55 years vs. 64 years, respectively). This should not significantly influence results, as EBV seroconversion occurs already in adolescence in the vast majority of patients [34].

Three out of ten EBV positive samples using PCR were confirmed in a subsequent ISH analysis. EBV infected lymphocytes is known to invade tumor tissue and thus the findings by PCR could be a result of tissue inflammation rather than the presence of viral gene products in tumor cells [35]. All three positive signals originated from stromal cells as well as from plasma. This is indicative that EBV genome was in fact present in the tumor cells.

However, in seven samples, EBV positivity by PCR could not be confirmed by ISH. The explanation could be the amplification of the target DNA in PCR based methods, resulting in a higher sensitivity than in ISH.

Another explanation could be that the ISH analysis was carried out using tissue slides cut subsequently to the original PCR tissue samples. This could influence the results as EBV infected cells can cluster inside the tumors, as described in more detail below [36].

Very few previous studies have examined the association between EOC and EBV and CMV. A serologic study reported a higher risk of ovarian cancer among patients with a late debut of mononucleosis, arguing that a late debut is a surrogate for a more severe clinical course. The same study found that elevated IgG titers to EBV viral capsid antigen were associated with a 5.3-fold (95% CI 1.5–18.4) increase in EOC risk, indicating that previous EBV infection could be associated with EOC [37]. In contrast, a recent serologic study reported no association between EOC and EBV antibody levels [38]. The same was true in a study by Khoury et al., where no evidence of EBV DNA in 419 ovarian serous cystadenocarcinomas from the Cancer Genome Atlas was found [39].

The prevalence of CMV was very low in the present study. This is in line with the aforementioned study that did not find serologic evidence of CMV infection [38]. Thus, results do not support the findings in two previous small studies. One study included 24 fresh malignant ovarian tissue samples and reported a prevalence of 50% by a PCR-based assay using CMV glycoprotein B gene-specific primers [40]. The other study detected human CMV tegument protein in 80% of cases using FFPE tissue samples from 10 patients with serous EOC [41].

The variance in the reported prevalence of CMV and EBV could be due to a number of factors. First, EBV and CMV seroprevalence is unevenly distributed among geographic regions [42, 43]. Second, differences in the samples studied and in the methodological approaches could play a role. Another explanation is tumor heterogeneity and the potential uneven distribution of infected cells. Arbach et al. demonstrated by PCR analysis that biopsies from the same malignant breast tumor could be EBV DNA positive as well as negative due to clustering of EBV-infected tumor cells [36].

Thirdly, most of the previous studies did not account for the number of patients who received neoadjuvant chemotherapy prior to specimen sampling. Chemotherapy could potentially increase the number of positive results due to reactivation of latent viral infection [41, 44]. In the present study, patients who received NACT were excluded, which could result in the lower prevalence.

Although our findings in this study were statistically significant, the EBV DNA prevalence was only 5% and only three samples were confirmed by ISH. This is considerably lower than the reported 80–100% prevalence of EBV in nasopharyngeal carcinoma tissue [45]. For other potential EBV-related cancers, prevalence ranges from 6 to 95%, with a higher incidence in Asian countries and among males [45]. The low EBV DNA prevalence in our study prompts caution in the interpretation of results. However, a low prevalence does not exclude the notion of viral initiation or promotion of tumorigenesis and then subsequent clearance from the host, also described as the “hit and run” hypothesis [46].

Moreover, inflammation is a common trait in some of the factors suspected of predisposing to EOC, including endometriosis, ovulation, and PID. Several studies demonstrate the important role of inflammatory mediators, such as interleukins and TNF alpha to the EOC microenvironment [47–49]. It is also plausible that more direct tumor-promoting actions could be imposed by specific infectious agents. A range of viral oncogenes can incapacitate key host proteins, enabling immune evasion, inhibition of apoptosis, and uncontrollable cell division [50, 51]. However, bacteria involved in PID can display similar effects, as studies have demonstrated that *Chlamydia trachomatis* can downregulate the DNA damage response and cause degradation of P53 [52, 53]. Furthermore, a recent study found that *Chlamydia trachomatis* was associated with increased EOC risk [38]. Finally, some of the factors that reduce EOC risk, including tubal ligation and hysterectomy, abrupt the passage to the pelvic cavity. Thus, they could impede microbiological invasion to the internal genitals and the suspected site of the precursor STIC lesions in the tubal fimbria. In this study, all EBV-positive samples were from serous EOC subtypes. This could be coincidental, but it could also indicate that a potential association between EOC and EBV infection is subtype specific.

## Conclusion

A significantly higher prevalence of EBV DNA was detected in EOC samples than in tissue samples from a benign control group. EBV infection is a contributing factor in other epithelial cancers, but more research is needed to uncover the potential relation to EOC.

## Additional files

Additional file 1: Data infect agents cancer. (DOCX 39 kb)

## Abbreviations

CMV: Cytomegalovirus
EBV: Epstein-Barr virus
EOC: Epithelial ovarian cancer
FFPE: Formalin-fixed and paraffin-embedded
FIGO: International Federation of Gynecology and Obstetrics
ISH: In-situ hybridization
NACT: Neoadjuvant Chemotherapy
PCR: Polymerase chain reaction
PID: Pelvic inflammatory disease
