# Supplementary material for: The prevalence of EBV and CMV DNA in epithelial ovarian cancer
Source: Infect Agent Cancer. 2019 Feb 26;14:7. doi: 10.1186/s13027-019-0223-z (PMC6390605; doi:10.1186/s13027-019-0223-z)
Supplement: Supplementary file 1 — Data infect agents cancer. (DOCX 39 kb) [file 13027_2019_223_MOESM1_ESM.docx]

| **Sample ID** | **CMV approved** | **CMV Infection** | **EVB approved** | **EBV Infection** | **Date and Time** | **Nucleic Acid Conc.** | **Unit** | **A260** | **A280** | **260/280** | **260/230** | **Sample Type** | **Factor** |
| --- | --- | --- | --- | --- | --- | --- | --- | --- | --- | --- | --- | --- | --- |
| 600-772 | ja | 0 | ja | 0 | 02-12-2014 11:16:47 | 232 | ng/µl | 4,639 | 2,297 | 2,02 | 0,97 | DNA | 50 |
| 600-773 | ja | 0 | ja | 0 | 02-12-2014 11:17:42 | 221,7 | ng/µl | 4,433 | 2,133 | 2,08 | 0,97 | DNA | 50 |
| 600-774 | ja | 0 | ja | 0 | 02-12-2014 11:19:00 | 273 | ng/µl | 5,461 | 2,706 | 2,02 | 1,08 | DNA | 50 |
| 600-775 | ja | 0 | ja | 0 | 02-12-2014 11:19:47 | 225,9 | ng/µl | 4,518 | 2,264 | 2 | 1,12 | DNA | 50 |
| 600-776 | ja | 0 | ja | 0 | 02-12-2014 11:20:26 | 302,8 | ng/µl | 6,057 | 2,978 | 2,03 | 1,28 | DNA | 50 |
| 600-777 | ja | 0 | ja | 0 | 02-12-2014 11:21:08 | 269,2 | ng/µl | 5,384 | 2,687 | 2 | 1,22 | DNA | 50 |
| 600-778 | ja | 0 | ja | 0 | 02-12-2014 11:22:24 | 611,7 | ng/µl | 12,234 | 6,166 | 1,98 | 1,63 | DNA | 50 |
| 600-779 | ja | 0 | ja | ja CT 37.22 | 02-12-2014 11:23:05 | 134,1 | ng/µl | 2,682 | 1,325 | 2,02 | 0,8 | DNA | 50 |
| 600-780 | ja | 0 | ja | 0 | 02-12-2014 11:24:53 | 226,7 | ng/µl | 4,533 | 2,28 | 1,99 | 1,07 | DNA | 50 |
| 600-781 | ja | 0 | ja | 0 | 02-12-2014 11:25:46 | 319,3 | ng/µl | 6,387 | 3,213 | 1,99 | 1,25 | DNA | 50 |
| 600-782 | ja | 0 | ja | 0 | 02-12-2014 11:26:35 | 179,6 | ng/µl | 3,593 | 1,798 | 2 | 0,93 | DNA | 50 |
| 600-783 | ja | 0 | ja | 0 | 02-12-2014 11:27:23 | 289,3 | ng/µl | 5,786 | 2,938 | 1,97 | 1,22 | DNA | 50 |
| 600-784 | ja | 0 | ja | ja CT 34.71 | 02-12-2014 11:28:16 | 484,7 | ng/µl | 9,695 | 4,846 | 2 | 1,51 | DNA | 50 |
| 600-785 | ja | 0 | ja | 0 | 02-12-2014 13:56:31 | 241,1 | ng/µl | 4,823 | 2,38 | 2,03 | 1,07 | DNA | 50 |
| 600-802 | ja | 0 | ja | 0 | 02-12-2014 10:09:04 | 75,5 | ng/µl | 1,509 | 0,637 | 2,37 | 0,45 | DNA | 50 |
| 600-803 | ja | 0 | ja | 0 | 02-12-2014 10:12:18 | 89,4 | ng/µl | 1,789 | 0,823 | 2,17 | 0,5 | DNA | 50 |
| 600-804 | ja | 0 | ja | 0 | 02-12-2014 10:13:19 | 113,3 | ng/µl | 2,265 | 1,045 | 2,17 | 0,59 | DNA | 50 |
| 600-805 | ja | 0 | ja | 0 | 02-12-2014 10:14:17 | 220,4 | ng/µl | 4,408 | 2,153 | 2,05 | 1,05 | DNA | 50 |
| 600-806 | ja | 0 | ja | 0 | 02-12-2014 10:15:23 | 302,6 | ng/µl | 6,053 | 2,93 | 2,07 | 0,99 | DNA | 50 |
| 600-807 | ja | 0 | ja | 0 | 02-12-2014 10:16:25 | 265 | ng/µl | 5,3 | 2,691 | 1,97 | 1,04 | DNA | 50 |
| 600-808 | ja | 0 | ja | 0 | 02-12-2014 10:17:34 | 167,4 | ng/µl | 3,349 | 1,645 | 2,04 | 0,82 | DNA | 50 |
| 600-809 | ja | 0 | ja | 0 | 02-12-2014 10:18:27 | 230,9 | ng/µl | 4,618 | 2,278 | 2,03 | 0,91 | DNA | 50 |
| 600-810 | ja | 0 | ja | 0 | 02-12-2014 10:19:23 | 134,3 | ng/µl | 2,687 | 1,297 | 2,07 | 0,73 | DNA | 50 |
| 600-811 | ja | 0 | ja | 0 | 02-12-2014 10:20:20 | 239,7 | ng/µl | 4,793 | 2,364 | 2,03 | 0,75 | DNA | 50 |
| 600-812 | ja | 0 | ja | 0 | 02-12-2014 10:21:27 | 342,8 | ng/µl | 6,856 | 3,358 | 2,04 | 1,3 | DNA | 50 |
| 600-813 | ja | 0 | ja | 0 | 02-12-2014 10:22:26 | 230,8 | ng/µl | 4,615 | 2,247 | 2,05 | 1,01 | DNA | 50 |
| 600-814 | ja | 0 | ja | 0 | 02-12-2014 10:25:40 | 180,6 | ng/µl | 3,611 | 1,793 | 2,01 | 0,72 | DNA | 50 |
| 600-815 | ja | 0 | ja | 0 | 02-12-2014 10:26:32 | 117,2 | ng/µl | 2,345 | 1,058 | 2,22 | 0,66 | DNA | 50 |
| 600-816 | ja | 0 | ja | 0 | 02-12-2014 10:28:10 | 49,8 | ng/µl | 0,997 | 0,433 | 2,3 | 0,28 | DNA | 50 |
| 600-817 | ja | 0 | ja | 0 | 02-12-2014 10:29:18 | 264,5 | ng/µl | 5,289 | 2,558 | 2,07 | 0,98 | DNA | 50 |
| 600-818 | ja | 0 | ja | 0 | 02-12-2014 10:30:27 | 572,7 | ng/µl | 11,454 | 5,732 | 2 | 1,44 | DNA | 50 |
| 600-819 | ja | 0 | ja | 0 | 02-12-2014 10:31:25 | 373,7 | ng/µl | 7,475 | 3,633 | 2,06 | 1,2 | DNA | 50 |
| 600-820 | ja | 0 | ja | 0 | 02-12-2014 10:32:20 | 38,3 | ng/µl | 0,765 | 0,347 | 2,21 | 0,17 | DNA | 50 |
| 600-821 | ja | 0 | ja | 0 | 02-12-2014 10:33:34 | 256,4 | ng/µl | 5,127 | 2,607 | 1,97 | 0,89 | DNA | 50 |
| 600-822 | ja | 0 | ja | 0 | 02-12-2014 10:38:41 | 196,7 | ng/µl | 3,934 | 1,857 | 2,12 | 0,96 | DNA | 50 |
| 600-823 | ja | 0 | ja | ja CT 36.03 | 02-12-2014 10:39:38 | 346 | ng/µl | 6,92 | 3,379 | 2,05 | 1,28 | DNA | 50 |
| 600-824 | ja | 0 | ja | 0 | 02-12-2014 10:40:37 | 427 | ng/µl | 8,541 | 4,31 | 1,98 | 1,3 | DNA | 50 |
| 600-825 | ja | 0 | ja | 0 | 02-12-2014 10:41:23 | 339,4 | ng/µl | 6,788 | 3,449 | 1,97 | 1,1 | DNA | 50 |
| 600-826 | ja | 0 | ja | 0 | 02-12-2014 10:42:17 | 451,8 | ng/µl | 9,036 | 4,404 | 2,05 | 1,32 | DNA | 50 |
| 600-827 | ja | 0 | ja | 0 | 02-12-2014 10:43:17 | 188,7 | ng/µl | 3,774 | 1,839 | 2,05 | 0,73 | DNA | 50 |
| 600-828 | ja | 0 | ja | 0 | 02-12-2014 10:44:11 | 165 | ng/µl | 3,3 | 1,523 | 2,17 | 0,84 | DNA | 50 |
| 600-829 | ja | 0 | ja | 0 | 02-12-2014 10:45:23 | 253,2 | ng/µl | 5,064 | 2,491 | 2,03 | 0,97 | DNA | 50 |
| 600-830 | ja | 0 | ja | 0 | 02-12-2014 10:49:03 | 272,8 | ng/µl | 5,455 | 2,682 | 2,03 | 1,15 | DNA | 50 |
| 600-831 | ja | 0 | ja | 0 | 02-12-2014 10:49:58 | 283,9 | ng/µl | 5,678 | 2,818 | 2,01 | 0,9 | DNA | 50 |
| 600-832 | ja | 0 | ja | 0 | 02-12-2014 10:50:50 | 33,7 | ng/µl | 0,674 | 0,226 | 2,98 | 0,27 | DNA | 50 |
| 600-833 | ja | 0 | ja | 0 | 02-12-2014 10:51:51 | 278,9 | ng/µl | 5,578 | 2,811 | 1,98 | 1,14 | DNA | 50 |
| 600-834 | ja | 0 | ja | 0 | 02-12-2014 10:52:45 | 385,1 | ng/µl | 7,702 | 3,88 | 1,99 | 1,2 | DNA | 50 |
| 600-835 | ja | 0 | ja | 0 | 02-12-2014 10:53:37 | 394,8 | ng/µl | 7,895 | 3,997 | 1,98 | 1,21 | DNA | 50 |
| 600-836 | ja | 0 | ja | 0 | 02-12-2014 10:54:28 | 159,3 | ng/µl | 3,187 | 1,661 | 1,92 | 0,79 | DNA | 50 |
| 600-837 | ja | 0 | ja | 0 | 02-12-2014 10:55:21 | 178 | ng/µl | 3,559 | 1,698 | 2,1 | 0,82 | DNA | 50 |
| 600-838 | ja | 0 | ja | 0 | 02-12-2014 10:55:59 | 525,8 | ng/µl | 10,516 | 5,247 | 2 | 1,44 | DNA | 50 |
| 600-839 | ja | 0 | ja | 0 | 02-12-2014 10:56:37 | 168,1 | ng/µl | 3,362 | 1,651 | 2,04 | 0,31 | DNA | 50 |
| 600-840 | ja | ? (02nd run) | ja | 0 | 02-12-2014 10:57:35 | 555,4 | ng/µl | 11,109 | 5,524 | 2,01 | 1,53 | DNA | 50 |
| 600-841 | ja | 0 | ja | 0 | 02-12-2014 10:58:21 | 121,7 | ng/µl | 2,434 | 1,167 | 2,08 | 0,65 | DNA | 50 |
| 600-842 | ja | 0 | ja | 0 | 02-12-2014 10:59:05 | 522,7 | ng/µl | 10,454 | 5,403 | 1,93 | 1,36 | DNA | 50 |
| 600-843 | ja | 0 | ja | 0 | 02-12-2014 10:59:50 | 377,7 | ng/µl | 7,553 | 3,824 | 1,98 | 1,16 | DNA | 50 |
| 600-844 | ja | 0 | ja | 0 | 02-12-2014 11:00:35 | 234,7 | ng/µl | 4,693 | 2,318 | 2,03 | 0,92 | DNA | 50 |
| 600-845 | ja | 0 | ja | 0 | 02-12-2014 11:01:37 | 597,9 | ng/µl | 11,958 | 5,993 | 2 | 1,53 | DNA | 50 |
| 600-846 | ja | 0 | ja | 0 | 02-12-2014 11:04:33 | 67,9 | ng/µl | 1,358 | 0,606 | 2,24 | 0,39 | DNA | 50 |
| 600-847 | ja | 0 | ja | 0 | 02-12-2014 11:05:17 | 131,8 | ng/µl | 2,636 | 1,273 | 2,07 | 0,72 | DNA | 50 |
| 600-848 | ja | 0 | ja | 0 | 02-12-2014 11:06:07 | 240,7 | ng/µl | 4,813 | 2,466 | 1,95 | 0,43 | DNA | 50 |
| 600-849 | ja | 0 | ja | 0 | 02-12-2014 11:06:46 | 337 | ng/µl | 6,74 | 3,499 | 1,93 | 1,26 | DNA | 50 |
| 600-850 | ja | 0 | ja | 0 | 02-12-2014 11:07:33 | 158,8 | ng/µl | 3,177 | 1,535 | 2,07 | 0,83 | DNA | 50 |
| 600-851 | ja | 0 | ja | 0 | 02-12-2014 11:08:24 | 369,9 | ng/µl | 7,397 | 3,745 | 1,98 | 1,06 | DNA | 50 |
| 600-852 | ja | 0 | ja | 0 | 02-12-2014 11:09:14 | 220 | ng/µl | 4,399 | 2,172 | 2,03 | 1,06 | DNA | 50 |
| 600-853 | ja | 0 | ja | 0 | 02-12-2014 11:10:05 | 454,9 | ng/µl | 9,098 | 4,563 | 1,99 | 1,26 | DNA | 50 |
| 600-854 | ja | 0 | ja | 0 |  |  |  |  |  |  |  |  |  |
| 600-855 | ja | 0 | ja | ja CT 35.34 | 02-12-2014 11:12:08 | 496,1 | ng/µl | 9,923 | 4,963 | 2 | 1,43 | DNA | 50 |
| 600-856 | ja | 0 | ja | 0 | 02-12-2014 11:13:14 | 101,8 | ng/µl | 2,036 | 0,947 | 2,15 | 0,5 | DNA | 50 |
| 600-857 | ja | 0 | ja | 0 | 02-12-2014 11:13:58 | 51,5 | ng/µl | 1,029 | 0,415 | 2,48 | 0,35 | DNA | 50 |
| 600-858 | ja | 0 | ja | 0 | 02-12-2014 11:14:43 | 65,9 | ng/µl | 1,317 | 0,57 | 2,31 | 0,33 | DNA | 50 |
| 600-859 | ja | 0 | ja | 0 | 02-12-2014 11:15:37 | 84,9 | ng/µl | 1,698 | 0,757 | 2,24 | 0,58 | DNA | 50 |
| 600-861 | ja | 0 | ja | 0 | 01-12-2014 14:04 | 348,5 | ng/µl | 6,97 | 3,443 | 2,02 | 1,09 | DNA | 50 |
| 600-862 | ja | ? (02nd run) | ja | 0 | 01-12-2014 14:08:50 | 291,9 | ng/µl | 5,838 | 2,852 | 2,05 | 1,11 | DNA | 50 |
| 600-863 | ja | 0 | ja | 0 | 01-12-2014 14:13:20 | 267,6 | ng/µl | 5,352 | 2,649 | 2,02 | 1,08 | DNA | 50 |
| 600-864 | ja | 0 | ja | 0 | 01-12-2014 14:14:31 | 377,2 | ng/µl | 7,544 | 3,739 | 2,02 | 1,18 | DNA | 50 |
| 600-865 | ja | 0 | ja | 0 | 01-12-2014 14:15:19 | 190,8 | ng/µl | 3,817 | 1,792 | 2,13 | 0,88 | DNA | 50 |
| 600-866 | ja | 0 | ja | 0 | 01-12-2014 14:16:29 | 278,5 | ng/µl | 5,57 | 2,727 | 2,04 | 0,99 | DNA | 50 |
| 600-867 | ja | 0 | ja | 0 | 01-12-2014 14:17:38 | 211,9 | ng/µl | 4,238 | 2,177 | 1,95 | 0,9 | DNA | 50 |
| 600-868 | ja | 0 | ja | 0 | 01-12-2014 14:19:39 | 404,6 | ng/µl | 8,092 | 3,975 | 2,04 | 1,25 | DNA | 50 |
| 600-869 | ja | 0 | ja | 0 | 02-12-2014 09:11:57 | 9,9 | ng/µl | 0,198 | 0 | -1182,35 | 0,07 | DNA | 50 |
| 600-870 | ja | 0 | ja | 0 | 02-12-2014 09:18:06 | 343,9 | ng/µl | 6,878 | 3,395 | 2,03 | 1,26 | DNA | 50 |
| 600-871 | ja | 0 | ja | 0 | 02-12-2014 09:19:12 | 435,8 | ng/µl | 8,715 | 4,357 | 2 | 1,37 | DNA | 50 |
| 600-872 | ja | 0 | ja | 0 | 02-12-2014 09:20:18 | 338,3 | ng/µl | 6,767 | 3,337 | 2,03 | 1,18 | DNA | 50 |
| 600-873 | ja | 0 | ja | 0 | 02-12-2014 09:21:16 | 306,3 | ng/µl | 6,125 | 3,006 | 2,04 | 1,15 | DNA | 50 |
| 600-874 | ja | 0 | ja | 0 | 02-12-2014 09:22:19 | 419,8 | ng/µl | 8,396 | 4,19 | 2 | 1,27 | DNA | 50 |
| 600-875 | ja | 0 | ja | 0 | 02-12-2014 09:23:29 | 255,7 | ng/µl | 5,115 | 2,527 | 2,02 | 1 | DNA | 50 |
| 600-876 | ja | 0 | ja | 0 | 02-12-2014 09:24:36 | 429,5 | ng/µl | 8,589 | 4,285 | 2 | 1,3 | DNA | 50 |
| 600-877 | ja | 0 | ja | 0 | 02-12-2014 09:26:05 | 242,1 | ng/µl | 4,843 | 2,409 | 2,01 | 1,02 | DNA | 50 |
| 600-878 | ja | 0 | ja | 0 | 02-12-2014 09:27:11 | 206,3 | ng/µl | 4,127 | 2,058 | 2 | 0,87 | DNA | 50 |
| 600-879 | ja | 0 | ja | 0 | 02-12-2014 09:59:35 | 142,7 | ng/µl | 2,853 | 1,356 | 2,1 | 0,68 | DNA | 50 |
| 600-880 | ja | 0 | ja | 0 | 02-12-2014 10:00:37 | 166,8 | ng/µl | 3,337 | 1,574 | 2,12 | 0,77 | DNA | 50 |
| 600-881 | ja | 0 | ja | 0 | 02-12-2014 10:01:41 | 129,6 | ng/µl | 2,592 | 1,193 | 2,17 | 0,63 | DNA | 50 |
| 600-882 | ja | 0 | ja | 0 | 02-12-2014 10:02:53 | 388,6 | ng/µl | 7,772 | 3,85 | 2,02 | 1,25 | DNA | 50 |
| 600-883 | ja | 0 | ja | 0 | 02-12-2014 13:59:57 | 274,1 | ng/µl | 5,482 | 2,686 | 2,04 | 1,14 | DNA | 50 |
| 600-884 | ja | 0 | ja | 0 | 02-12-2014 14:00:38 | 127,3 | ng/µl | 2,545 | 1,223 | 2,08 | 0,74 | DNA | 50 |
| 600-885 | ja | 0 | ja | ja CT 33.30 | 02-12-2014 14:01:25 | 379,3 | ng/µl | 7,585 | 3,796 | 2 | 1,32 | DNA | 50 |
| 600-886 | ja | 0 | ja | 0 | 02-12-2014 14:02:03 | 177,4 | ng/µl | 3,549 | 1,751 | 2,03 | 0,91 | DNA | 50 |
| 600-887 | ja | 0 | ja | 0 | 02-12-2014 14:02:40 | 445,9 | ng/µl | 8,918 | 4,453 | 2 | 1,44 | DNA | 50 |
| 600-908 | ja | 0 | ja | 0 | 02-12-2014 12:43:58 | 448,4 | ng/µl | 8,969 | 4,511 | 1,99 | 1,38 | DNA | 50 |
| 600-909 | ja | 0 | ja | 0 | 02-12-2014 12:44:47 | 247,2 | ng/µl | 4,943 | 2,442 | 2,02 | 1,08 | DNA | 50 |
| 600-910 | ja | 0 | ja | 0 | 02-12-2014 12:45:32 | 217,4 | ng/µl | 4,349 | 2,218 | 1,96 | 0,95 | DNA | 50 |
| 600-911 | ja | 0 | ja | 0 | 02-12-2014 12:46:26 | 362,2 | ng/µl | 7,245 | 3,648 | 1,99 | 1,32 | DNA | 50 |
| 600-912 | ja | 0 | ja | 0 | 02-12-2014 12:48:04 | 60,4 | ng/µl | 1,209 | 0,413 | 2,93 | 0,38 | DNA | 50 |
| 600-913 | ja | 0 | ja | 0 | 02-12-2014 12:49:04 | 298 | ng/µl | 5,96 | 2,913 | 2,05 | 1,23 | DNA | 50 |
| 600-914 | ja | 0 | ja | 0 | 02-12-2014 12:50:06 | 290,6 | ng/µl | 5,813 | 2,942 | 1,98 | 1,11 | DNA | 50 |
| 600-915 | ja | 0 | ja | 0 | 02-12-2014 12:51:04 | 290 | ng/µl | 5,8 | 2,778 | 2,09 | 1,15 | DNA | 50 |
| 600-916 | ja | 0 | ja | 0 | 02-12-2014 12:52:15 | 239,2 | ng/µl | 4,785 | 2,358 | 2,03 | 1,04 | DNA | 50 |
| 600-917 | ja | 0 | ja | 0 | 02-12-2014 13:18:53 | 355,3 | ng/µl | 7,106 | 3,534 | 2,01 | 1,27 | DNA | 50 |
| 600-918 | ja | 0 | ja | 0 | 02-12-2014 13:19:41 | 421,6 | ng/µl | 8,431 | 4,188 | 2,01 | 1,39 | DNA | 50 |
| 600-919 | ja | 0 | ja | 0 | 02-12-2014 13:20:29 | 72,2 | ng/µl | 1,444 | 0,642 | 2,25 | 0,47 | DNA | 50 |
| 600-920 | ja | 0 | ja | 0 | 02-12-2014 13:21:10 | 164,9 | ng/µl | 3,299 | 1,598 | 2,06 | 0,87 | DNA | 50 |
| 600-921 | ja | 0 | ja | 0 | 02-12-2014 13:22:05 | 119,4 | ng/µl | 2,388 | 1,114 | 2,14 | 0,7 | DNA | 50 |
| 600-922 | ja | 0 | ja | 0 | 02-12-2014 13:22:57 | 395,8 | ng/µl | 7,915 | 4,038 | 1,96 | 1,37 | DNA | 50 |
| 600-923 | ja | 0 | ja | 0 | 02-12-2014 13:23:42 | 95,7 | ng/µl | 1,914 | 0,896 | 2,14 | 0,62 | DNA | 50 |
| 600-924 | ja | ? (02nd run) | ja | 0 | 02-12-2014 13:26:15 | 193,5 | ng/µl | 3,87 | 1,906 | 2,03 | 0,96 | DNA | 50 |
| 600-925 | ja | 0 | ja | 0 | 02-12-2014 13:27:05 | 1317,4 | ng/µl | 26,349 | 13,807 | 1,91 | 1,9 | DNA | 50 |
| 600-926 | ja | 0 | ja | 0 | 02-12-2014 13:27:54 | 410,6 | ng/µl | 8,212 | 3,993 | 2,06 | 1,37 | DNA | 50 |
| 600-927 | ja | 0 | ja | 0 | 02-12-2014 13:28:36 | 420,1 | ng/µl | 8,402 | 4,376 | 1,92 | 1,39 | DNA | 50 |
| 600-929 | ja | 0 | ja | 0 | 02-12-2014 13:29:24 | 445,7 | ng/µl | 8,913 | 4,418 | 2,02 | 1,36 | DNA | 50 |
| 600-930 | ja | 0 | ja | 0 | 02-12-2014 13:30:19 | 36,1 | ng/µl | 0,722 | 0,274 | 2,63 | 0,28 | DNA | 50 |
| 600-931 | ja | 0 | ja | 0 | 02-12-2014 13:31:14 | 141,2 | ng/µl | 2,824 | 1,301 | 2,17 | 0,76 | DNA | 50 |
| 600-932 | ja | 0 | ja | 0 | 02-12-2014 13:31:57 | 433,3 | ng/µl | 8,666 | 4,275 | 2,03 | 1,41 | DNA | 50 |
| 600-933 | ja | 0 | ja | 0 | 02-12-2014 13:57:12 | 479,2 | ng/µl | 9,584 | 4,692 | 2,04 | 1,46 | DNA | 50 |
| 600-935 | ja | 0 | ja | 0 | 02-12-2014 13:58:15 | 216,9 | ng/µl | 4,338 | 2,265 | 1,92 | 0,95 | DNA | 50 |
| 600-944 | ja | 0 | ja | 0 | 02-12-2014 13:32:47 | 927,9 | ng/µl | 18,558 | 9,314 | 1,99 | 1,81 | DNA | 50 |
| 600-955 | ja | 0 | ja | ja CT 35.20 | 02-12-2014 09:32:55 | 355,1 | ng/µl | 7,102 | 3,535 | 2,01 | 1,24 | DNA | 50 |
| 600-956 | ja | 0 | ja | 0 | 02-12-2014 09:34:18 | 310,8 | ng/µl | 6,217 | 3,092 | 2,01 | 1,17 | DNA | 50 |
| 600-957 | ja | 0 | ja | 0 | 02-12-2014 09:35:21 | 352,2 | ng/µl | 7,043 | 3,56 | 1,98 | 1,22 | DNA | 50 |
| 600-958 | ja | 0 | ja | 0 | 02-12-2014 09:36:11 | 235,5 | ng/µl | 4,71 | 2,295 | 2,05 | 0,91 | DNA | 50 |
| 600-959 | ja | 0 | ja | ja CT 35.92 | 02-12-2014 09:37:04 | 875,6 | ng/µl | 17,512 | 8,819 | 1,99 | 1,7 | DNA | 50 |
| 600-960 | ja | 0 | ja | 0 | 02-12-2014 09:38:16 | 304 | ng/µl | 6,08 | 3,009 | 2,02 | 1,12 | DNA | 50 |
| 600-961 | ja | 0 | ja | 0 | 02-12-2014 09:39:16 | 441,1 | ng/µl | 8,822 | 4,376 | 2,02 | 1,34 | DNA | 50 |
| 600-962 | ja | 0 | ja | 0 | 02-12-2014 09:40:25 | 170,3 | ng/µl | 3,406 | 1,705 | 2 | 0,75 | DNA | 50 |
| 600-963 | ja | 0 | ja | 0 | 02-12-2014 09:41:20 | 350 | ng/µl | 7 | 3,474 | 2,01 | 1,25 | DNA | 50 |
| 600-964 | ja | 0 | ja | 0 | 02-12-2014 09:42:23 | 317,8 | ng/µl | 6,355 | 3,15 | 2,02 | 1,21 | DNA | 50 |
| 600-965 | ja | 0 | ja | 0 | 02-12-2014 09:43:30 | 535,1 | ng/µl | 10,702 | 5,393 | 1,98 | 1,45 | DNA | 50 |
| 600-966 | ja | 0 | ja | 0 | 02-12-2014 09:44:45 | 415,2 | ng/µl | 8,304 | 4,107 | 2,02 | 1,33 | DNA | 50 |
| 600-967 | ja | 0 | ja | 0 | 02-12-2014 09:46:14 | 224,1 | ng/µl | 4,482 | 2,213 | 2,02 | 0,94 | DNA | 50 |
| 600-968 | ja | 0 | ja | 0 | 02-12-2014 09:47:45 | 732,8 | ng/µl | 14,656 | 7,403 | 1,98 | 1,59 | DNA | 50 |
| 600-969 | ja | 0 | ja | 0 | 02-12-2014 09:48:41 | 252,1 | ng/µl | 5,042 | 2,482 | 2,03 | 0,99 | DNA | 50 |
| 600-970 | ja | 0 | ja | 0 | 02-12-2014 09:56:25 | 308,8 | ng/µl | 6,177 | 3,113 | 1,98 | 1,18 | DNA | 50 |
| 600-971 | ja | 0 | ja | 0 | 02-12-2014 09:57:24 | 440,3 | ng/µl | 8,806 | 4,388 | 2,01 | 1,34 | DNA | 50 |
| 600-972 | ja | 0 | ja | 0 | 02-12-2014 09:58:13 | 289,1 | ng/µl | 5,782 | 2,884 | 2 | 1 | DNA | 50 |
| 600-973 | ja | 0 | ja | 0 | 02-12-2014 13:33:37 | 544,7 | ng/µl | 10,894 | 5,378 | 2,03 | 1,52 | DNA | 50 |
| 600-974 | ja | 0 | ja | 0 | 02-12-2014 13:34:36 | 429,4 | ng/µl | 8,589 | 4,366 | 1,97 | 1,41 | DNA | 50 |
| 600-975 | ja | 0 | ja | 0 | 02-12-2014 13:35:20 | 365,9 | ng/µl | 7,318 | 3,607 | 2,03 | 1,31 | DNA | 50 |
| 600-976 | ja | 0 | ja | 0 |  |  |  |  |  |  |  |  |  |
| 600-977 | ja | 0 | ja | 0 | 02-12-2014 13:36:33 | 428 | ng/µl | 8,559 | 4,335 | 1,97 | 1,4 | DNA | 50 |
| 600-978 | ja | 0 | ja | 0 | 02-12-2014 13:37:20 | 740,8 | ng/µl | 14,816 | 7,647 | 1,94 | 1,48 | DNA | 50 |
| 600-979 | ja | 0 | ja | 0 | 02-12-2014 13:38:08 | 512,5 | ng/µl | 10,249 | 5,179 | 1,98 | 1,49 | DNA | 50 |
| 600-980 | ja | 0 | ja | 0 | 02-12-2014 13:38:48 | 313,1 | ng/µl | 6,262 | 3,144 | 1,99 | 1,15 | DNA | 50 |
| 600-981 | ja | 0 | ja | 0 | 02-12-2014 13:39:41 | 200,2 | ng/µl | 4,004 | 1,925 | 2,08 | 0,99 | DNA | 50 |
| 600-982 | ja | 0 | ja | 0 | 02-12-2014 13:40:27 | 546,3 | ng/µl | 10,926 | 5,439 | 2,01 | 1,54 | DNA | 50 |
| 600-983 | ja | 0 | ja | 0 | 02-12-2014 13:41:18 | 96,9 | ng/µl | 1,939 | 0,869 | 2,23 | 0,61 | DNA | 50 |
| 600-984 | ja | 0 | ja | 0 | 02-12-2014 13:43:16 | 619,7 | ng/µl | 12,394 | 6,231 | 1,99 | 1,61 | DNA | 50 |
| 600-985 | ja | 0 | ja | 0 | 02-12-2014 13:43:58 | 62,6 | ng/µl | 1,252 | 0,494 | 2,54 | 0,3 | DNA | 50 |
| 600-986 | ja | 0 | ja | 0 | 02-12-2014 13:44:55 | 82,2 | ng/µl | 1,645 | 0,756 | 2,17 | 0,4 | DNA | 50 |
| 600-987 | ja | 0 | ja | 0 | 02-12-2014 13:45:34 | 118,5 | ng/µl | 2,369 | 1,12 | 2,12 | 0,68 | DNA | 50 |
| 600-988 | ja | 0 | ja | 0 | 02-12-2014 13:46:20 | 351,2 | ng/µl | 7,023 | 3,528 | 1,99 | 1,29 | DNA | 50 |
| 600-989 | ja | 0 | ja | 0 | 02-12-2014 13:46:59 | 307 | ng/µl | 6,14 | 3,175 | 1,93 | 1,23 | DNA | 50 |
| 600-990 | ja | 0 | ja | ja CT 35.88 | 02-12-2014 13:47:39 | 389,1 | ng/µl | 7,783 | 3,897 | 2 | 1,36 | DNA | 50 |
| 600-991 | ja | 0 | ja | 0 | 02-12-2014 13:48:17 | 395,4 | ng/µl | 7,908 | 3,954 | 2 | 1,37 | DNA | 50 |
| 600-992 | ja | 0 | ja | 0 | 02-12-2014 13:54:19 | 494,5 | ng/µl | 9,891 | 4,948 | 2 | 1,47 | DNA | 50 |
| 600-993 | ja | 0 | ja | 0 | 02-12-2014 13:49:10 | 412,4 | ng/µl | 8,249 | 4,121 | 2 | 1,38 | DNA | 50 |
| 600-994 | ja | 0 | ja | 0 | 02-12-2014 13:49:56 | 388,9 | ng/µl | 7,779 | 3,849 | 2,02 | 1,36 | DNA | 50 |
| 600-995 | ja | 0 | ja | 0 | 02-12-2014 13:50:34 | 437,8 | ng/µl | 8,756 | 4,317 | 2,03 | 1,41 | DNA | 50 |
| 600-996 | ja | 0 | ja | 0 | 02-12-2014 13:51:09 | 251 | ng/µl | 5,02 | 2,559 | 1,96 | 1,12 | DNA | 50 |
| 600-997 | ja | 0 | ja | 0 | 02-12-2014 13:51:41 | 186,5 | ng/µl | 3,73 | 1,779 | 2,1 | 0,95 | DNA | 50 |
| 600-998 | ja | 0 | ja | 0 | 02-12-2014 13:52:19 | 308,8 | ng/µl | 6,175 | 3,062 | 2,02 | 1,2 | DNA | 50 |
| 600-999 | ja | 0 | ja | 0 | 02-12-2014 13:53:18 | 632,3 | ng/µl | 12,646 | 6,362 | 1,99 | 1,54 | DNA | 50 |
| 601-000 | ja | 0 | ja | 0 | 02-12-2014 14:05:36 | 315,7 | ng/µl | 6,314 | 3,139 | 2,01 | 1,21 | DNA | 50 |
| 601-001 | ja | 0 | ja | 0 | 02-12-2014 14:07:25 | 376 | ng/µl | 7,521 | 3,745 | 2,01 | 0,98 | DNA | 50 |
| 601-002 | ja | 0 | ja | 0 | 02-12-2014 14:07:58 | 322,4 | ng/µl | 6,448 | 3,168 | 2,04 | 1,25 | DNA | 50 |
| 601-003 | ja | 0 | ja | 0 | 02-12-2014 14:08:34 | 342,9 | ng/µl | 6,859 | 3,472 | 1,98 | 1,19 | DNA | 50 |
| 601-004 | ja | 0 | ja | 0 | 02-12-2014 14:09:10 | 140,3 | ng/µl | 2,806 | 1,326 | 2,12 | 0,76 | DNA | 50 |
| 601-005 | ja | 0 | ja | 0 | 02-12-2014 14:09:45 | 175,6 | ng/µl | 3,512 | 1,683 | 2,09 | 0,92 | DNA | 50 |
| 601-006 | ja | 0 | ja | 0 | 02-12-2014 14:10:18 | 356,8 | ng/µl | 7,136 | 3,505 | 2,04 | 1,28 | DNA | 50 |
| 601-007 | ja | 0 | ja | 0 | 02-12-2014 14:10:55 | 406,2 | ng/µl | 8,124 | 4,068 | 2 | 1,39 | DNA | 50 |
| 601-008 | ja | 0 | ja | 0 |  |  | ng/µl |  |  |  |  | DNA | 50 |
| 601-009 | ja | 0 | ja | 0 |  |  | ng/µl |  |  |  |  | DNA | 50 |
| 601-010 | ja | 0 | ja | 0 |  |  | ng/µl |  |  |  |  | DNA | 50 |
| 601-011 | ja | 0 | ja | 0 |  |  | ng/µl |  |  |  |  | DNA | 50 |
| 601-012 | ja | 0 | ja | 0 |  |  | ng/µl |  |  |  |  | DNA | 50 |
| 601-013 | ja | 0 | ja | 0 |  |  | ng/µl |  |  |  |  | DNA | 50 |
| 601-015 | ja | 0 | ja | 0 |  |  | ng/µl |  |  |  |  | DNA | 50 |
| 601-016 | ja | 0 | ja | ja CT 33.73 |  |  | ng/µl |  |  |  |  | DNA | 50 |
| 601-017 | ja | 0 | ja | 0 |  |  | ng/µl |  |  |  |  | DNA | 50 |
| 601-018 | ja | 0 | ja | 0 |  |  | ng/µl |  |  |  |  | DNA | 50 |
| 601-019 | ja | 0 | ja | 0 |  |  | ng/µl |  |  |  |  | DNA | 50 |
| 601-020 | ja | 0 | ja | 0 |  |  | ng/µl |  |  |  |  | DNA | 50 |
| 601-021 | ja | 0 | ja | 0 |  |  | ng/µl |  |  |  |  | DNA | 50 |
| 601-022 | ja | 0 | ja | 0 |  |  | ng/µl |  |  |  |  | DNA | 50 |
| 601-023 | ja | 0 | ja | 0 |  |  | ng/µl |  |  |  |  | DNA | 50 |
| 601-118 | ja | 0 | ja | 0 | 02-12-2014 14:11:31 | 396,6 | ng/µl | 7,931 | 3,95 | 2,01 | 1,33 | DNA | 50 |
| 601-119 | ja | 0 | ja | 0 | 02-12-2014 14:12:12 | 261,4 | ng/µl | 5,229 | 2,59 | 2,02 | 1,09 | DNA | 50 |
| 601-120 | ja | 0 | ja | 0 | 02-12-2014 14:12:48 | 335,5 | ng/µl | 6,711 | 3,324 | 2,02 | 1,17 | DNA | 50 |
| 601-121 | ja | 0 | ja | ja CT 34.16 | 02-12-2014 14:13:26 | 383,1 | ng/µl | 7,663 | 3,857 | 1,99 | 1,36 | DNA | 50 |
